# Risk factors associated with late hepatocellular carcinoma detection in patients undergoing regular surveillance

**DOI:** 10.1097/MD.0000000000034637

**Published:** 2023-08-11

**Authors:** Sangmi Jang, Young-Joo Jin, Jin-Woo Lee, Dam Kwon, Jung Hwan Yu

**Affiliations:** a Digestive Disease Center, Department of Internal Medicine, Inha University Hospital, Inha University School of Medicine, Incheon, South Korea.

**Keywords:** advanced stage, child-pugh score, hepatocellular carcinoma, surveillance

## Abstract

Hepatocellular carcinoma (HCC) has a very poor prognosis with a 5-year survival rate of < 20%; hence, early diagnosis is crucial. Despite regular checkups for high-risk groups of HCC, there are a few cases in which it is discovered as a late-stage HCC. Therefore, this study aimed to investigate the characteristics of patients with delayed HCC detection during regular surveillance. Between January 2010 and December 2020, we analyzed patients with newly diagnosed HCCs who underwent HCC surveillance by ultrasound or computed tomography scan at least twice and were followed up for more than 1 year for hepatitis B, hepatitis C, and chronic liver disease. The mean age of 223 HCC patients was 70 years, of which 152 were male, accounting for 68.1%. Among them, 196 patients (87%) were diagnosed with Barcelona clinic liver cancer stage 0 or A, while 27 (13%) were diagnosed with Barcelona clinic liver cancer stages B and C. When classified according to the TNM criteria, 154 patients (69%) were in stage I, and 69 (31%) were in stage II or higher. Multivariate analysis was performed to identify the risk factors for patients diagnosed with late-stage HCC. The Child–Turcotte–Pugh (CTP) score was identified as a highly significant factor (*P* = .002, HR 1.547, 95% CI 1.177–2.032), whereas the presence of cirrhosis, body mass index, and sex had no significant effect. We found that in patients with chronic liver disease who were screened regularly, those with higher CTP scores were more likely to be diagnosed with HCC in the late-stages. Therefore, although the presence of cirrhosis is also important for HCC surveillance, careful attention is needed in patients with high CTP scores.

## 1. Introduction

Hepatocellular carcinoma (HCC) is the 6th most common cancer worldwide and a leading cause of death in patients with liver cirrhosis.^[1]^ HCC has a high incidence rate globally, and the incidence is increasing worldwide. Previously, the annual incidence of HCC ranged from 2 to 7 per 100,000 people worldwide in the early 21^st^ century.^[2]^ More recent data show that there were 9.3 new cases of liver cancer worldwide for every 100,000 people in 2018, while the mortality rate was 8.5, according to estimates.^[3]^ Moreover, approximately 747,000 HCC cases were reported globally in 2019, showing a 70% increase in HCC incidence since 1990, and HCC was also responsible for 480,000 deaths.^[4]^ HCC has a very poor prognosis, with a 5-year survival rate of <20%. The Barcelona clinic liver cancer (BCLC) staging system,^[5]^ proposed by the European Association for the Study of the Liver (EASL), is an algorithm that shows appropriate options for managing HCC at various stages. Potentially curative therapies, including liver transplantation, resection, or radiofrequency ablation, are available only for patients with very early- to early-stage HCC. However, patients with indeterminate or advanced stage HCCs account for 40% to 50% of all diagnoses. Patients with late-stage HCC have a limited prognosis and a median survival of 11 to 20 months because only palliative treatment options are available.^[6]^

Patients in the late-stages of HCC, such as intermediate, advanced, and terminal stages, have unresectable HCCs and do not usually have the chance of receiving curative treatment. According to the BCLC criteria, the indeterminate stage is defined as BCLC stage B, with multinodular tumors with preserved liver function and an Eastern Cooperative Oncology Group performance status of 0. An advanced stage is defined as BCLC stage C with preserved liver function, an Eastern Cooperative Oncology Group performance status of 1 to 2, portal invasion, and extrahepatic metastasis.^[5]^ Early diagnosis of HCC is essential because patients diagnosed with late-stage HCC cannot receive curative treatment and have a very poor prognosis. Patients with a high-risk of HCC development should undergo HCC surveillance, including radiological examinations such as ultrasonography.^[7]^ The European Association for the Study of the Liver guidelines for HCC surveillance recommend that patients classified as high-risk for developing HCC undergo HCC surveillance by abdominal ultrasound every 6 months.^[8]^ However, despite the effort of regular checkups and close monitoring of patients at high-risk for HCC, there are still a few cases in which HCCs are detected at intermediate or advanced stages.

Therefore, this study examined the characteristics of patients with delayed HCC detection during regular HCC surveillance to define and understand the patient group at a higher risk for developing late-stage HCC.

## 2. Materials and methods

### 2.1. Study subjects

Between January 2010 and December 2020, 2960 patients with newly diagnosed HCCs were retrieved from the Clinical Data Warehouse at Inha University Hospital by searching the International Classification of Diseases-10 code C220. The patients were > 18 years old and were diagnosed with chronic hepatitis B, chronic hepatitis C, or other chronic liver diseases, including nonalcoholic steatohepatitis (NASH), alcoholic liver disease, and cryptogenic liver cirrhosis. The patients underwent HCC surveillance using ultrasound or computed tomography (CT) scans at least twice during the study period and were followed up for more than 1 year. HCC was diagnosed by histopathology or conventional imaging tests according to the Korean Liver Cancer Guidelines,^[9]^ and HCC staging was determined by the BCLC classification and the 8^th^ edition of the American Joint Committee on Cancer (AJCC)-TNM classification.^[10]^

**Figure 1.**
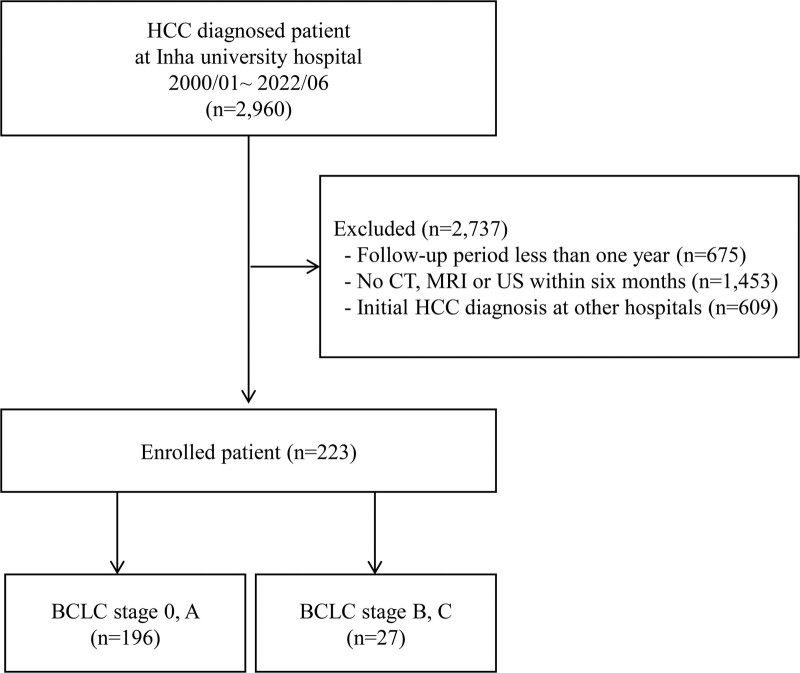
Flow chart of the patients. Of the 2960 patients with newly diagnosed HCCs at our institution from January 2010 to December 2020, 223 patients with HCC diagnosed at our hospital were enrolled in this study. 196 (87%) patients were diagnosed with BCLC stages 0 and A, while 27 (13%) patients were diagnosed with advanced stages of BCLC stages B and C. BCLC = Barcelona clinic liver cancer, HCC = hepatocellular carcinoma.

Patients with a follow-up period of less than 1 year (n = 675), patients without imaging tests of CT, liver magnetic resonance imaging, or ultrasonography within 6 months (n = 1453), and patients who were initially diagnosed with HCC at other centers (n = 609) were excluded. Patients diagnosed with autoimmune hepatitis, primary biliary cirrhosis, or Wilson’s disease were also excluded. Therefore, 223 patients diagnosed with de novo HCCs were enrolled in this study (Fig. 1).

This study was conducted in compliance with the guidelines of the Helsinki Declaration and was approved by the institutional review board of Inha University Hospital, Incheon, South Korea (IRB approval number: INHAUH 2023-04-028). The requirement for informed consent was waived by the IRB owing to the retrospective nature of the study.

### 2.2. Data collection

Clinical and laboratory data were collected by retrieving information from the Clinical Data Warehouse and reviewing medical records. Hypertension was defined as a systolic blood pressure > 140 mm Hg, a diastolic blood pressure > 90 mm Hg, or a history of antihypertensive medication prescription. Diabetes was defined as a fasting glucose level > 126 mg/dL, a random glucose level > 200 mg/dL, or a past prescription for antidiabetic drugs. Hyperlipidemia was defined as a total cholesterol level of < 200 mg/dL or a history of taking antihyperlipidemic medication.^[11]^ For men and women, significant alcohol intake was defined as 30 g/day and 20 g/day, respectively.^[12]^

White blood cell (WBC) count, hemoglobin, platelets, albumin, total bilirubin, alkaline phosphatase, aspartate aminotransferase (AST), alanine aminotransferase (ALT), glucose, total cholesterol, and alpha-fetoprotein (AFP) levels were collected. The Child–Turcotte–Pugh (CTP) score was calculated according to the patient’s albumin level, total bilirubin level, prothrombin time (PT) international ratio (INR), presence of ascites, and hepatic encephalopathy.

### 2.3. Clinical characteristics comparison according to the HCC stages

This study analyzed patients diagnosed with de novo HCC according to 2 different staging systems: BCLC staging of 0, A, B, and C and TNM staging of I through IV according to the AJCC 8^th^ edition staging system for HCC.^[10]^ Patients in BCLC stages 0 and A were compared with patients in BCLC stages B and C, while patients in AJCC-TNM stage I were compared with patients in stages II, III, and IV.

### 2.4. Statistical analysis

Depending on the normality of the data distribution, various clinical and laboratory variables are presented as the mean ± standard deviation, or median (interquartile range, IQR). The Student *t* test was used for continuous variables, and Fisher’s exact test was used for categorical variables. *P* values < .05 were considered significant. SPSS software (Windows version 25.0; SPSS Inc., Chicago, IL) and R (Windows version 4.0.5) were used to conduct statistical analyses.

## 3. Results

### 3.1. Baseline clinical characteristics of the patients with de novo HCC according to BCLC staging

**Figure 2.**
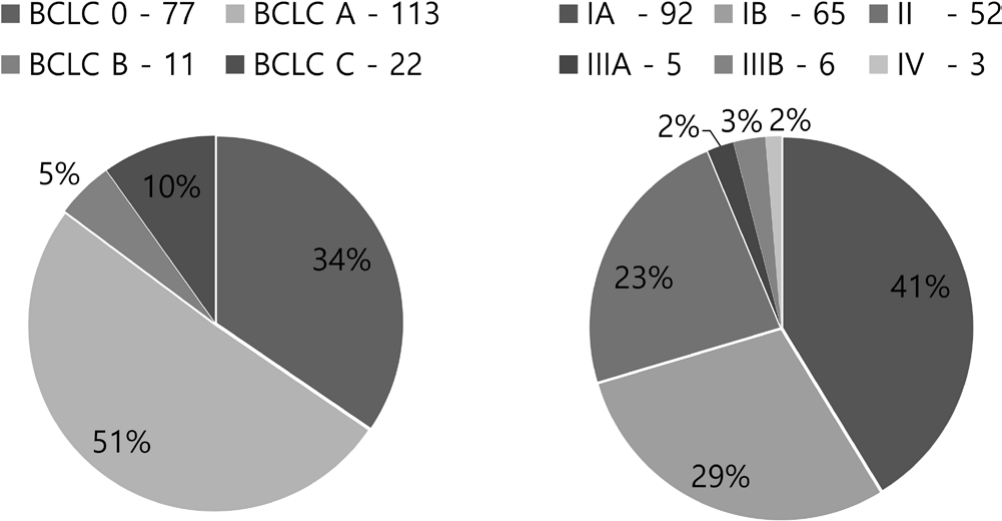
BCLC and TNM classification of the HCC patients. According to the BCLC staging, 77 (34%) HCC patients were in BCLC stage 0, 113 (51%) patients were in BCLC stage A, 11 (5%) patients were in BCLC stage B, and 22 (10%) patients were in BCLC stage C. According to the AJCC-TNM staging, 92 (41%) HCC patients were in AJCC-TNM stage IA, 65 (29%) patients in AJCC-TNM stage IB, 52 (23%) patients in AJCC-TNM stage II, 5 (2%) patients in AJCC-TNM stage IIIA, 6 (3%) patients in AJCC-TNM stage IIIB, and 3 (1%) patients in AJCC-TNM stage IV. AJCC = American joint committee on cancer, BCLC = Barcelona clinic liver cancer, HCC = hepatocellular carcinoma.

Table 1 lists the comparative findings of clinical features at diagnosis. The mean age of the 223 HCC patients was 70.0 years, of which 152 were male, accounting for 68.1%. Among these patients, 196 patients (87%) were diagnosed with BCLC stages 0 and A, while 27 patients (13%) were in the late-stages of BCLC stages B and C. There were 77 (34%), 113 (51%), 11 (5%), and 22 patients (10%) with HCC in BCLC stage 0, BCLC stage A, BCLC stage B, and BCLC stage C, respectively (Fig. 2).

**Table 1:** Baseline clinical characteristics of the study subjects (BCLC stage).

Variables	Total (n = 223)	BCLC stage 0, A (n = 196, 87%)	BCLC stage B, C (n = 27, 13%)	*P* value*
Age (yr)†	70.0 (29.0–98.0)	69.7 (28.0–98.0)	72.0 (57.0–92.0)	.264
Sex (male), n (%)	152 (68.1)	132 (67.3)	20 (74.0)	.482
DM, n (%)	72 (32.2)	68 (34.6)	4 (14.8)	.038
Hypertension, n (%)	63 (28.2)	57 (29.0)	6 (22.2)	.458
LC, n (%)	167 (74.9)	146 (74.4)	21 (77.7)	.712
Fatty liver, n (%)	12 (5.3)	12 (6.1)	0 (0.0)	.186
CTP A/B/C n (%)	186/29/8 (83.4/13.0/3.6)	169/23/4 (86.1/11.7/0.2)	17/6/4 (63.0/22.2/14.8)	.001
BMI (kg/m^2^)	24.7 (15.4–35.9)	24.9 (15.4–35.9)	23.3 (17.0–34.8)	.760
LS (kPa)	20.8 (4.0–75.0)	21.4 (4.0–75.0)	6.4 (4.0–12.0)	.145
CAP (dB/m)	226.5 (125.0–337.0)	227.1 (125.0–337.0)	207.0 (207.0–207.0)	.728
WBC (1000/*µℓ*)	4696 (1600–10,030)	4670 (1600–9480)	4650 (2360–10,000)	.903
Hemoglobin (g/dL)	13.1 (6.4–17.7)	13.2 (6.4–17.7)	12.1 (8.5–15.8)	.020
Platelet (1000/*µℓ*)	111.6 (24.0–334.0)	111.8 (24.0–334.0)	107.9 (28.0–287.0)	.460
AFP (ng/mL)	22.0 (0.9–493.0)	22.2 (0.9–493.0)	19.9 (2.0–145.0)	.840
AST (IU/L)	52.6 (15.0–441.0)	50.5 (18.0–411.0)	67.6 (15.0–441.0)	.780
ALT (IU/L)	43.2 (5.0–511.0)	42.5 (5.0–511.0)	48.3 (10.0–298.0)	.537
Total bilirubin (mg/dL)	1.3 (0.2–5.3)	1.2 (0.2–4.5)	1.6 (0.3–5.3)	.008
Albumin (g/dL)	3.7 (2.0–5.2)	3.8 (2.0–5.2)	3.3 (2.3–4.9)	.001
Creatinine (mg/dL)	0.9 (0.5–3.5)	0.9 (0.5–3.5)	0.92 (0.7–1.5)	.829
PT, INR	1.2 (0.9–2.0)	1.2 (0.9–2.0)	1.3 (0.9–1.8)	.004
Total cholesterol (mg/dL)	152.1 (52.0–585.0)	154.5 (64.0–585.0)	135.4 (52.0–270.0)	.061

AFP = alpha-fetoprotein, ALT = alanine aminotransferase, AST = aspartate aminotransferase, BMI = body mass index, BCLC = Barcelona clinic liver cancer, CAP = controlled attenuation parameter, CTP = Child–Turcotte–Pugh, DM = diabetes mellitus, INR = international ratio, LC = liver cirrhosis, LS = liver stiffness, PT = prothrombin time, WBC = white blood cell.

* *P* values were calculated using the *t* test or *chi*-square test.

† Median (range).

There were no significant differences in the median age (69.7 vs 72.0 years, *P* = .264) or proportion of male patients (67.3% vs 74%, *P* = .482) between the BCLC stage 0/A group and the BCLC stage B/C group. The BCLC stage 0/A group showed a higher hemoglobin level (13.2 vs 12.1 g/dL, *P* = .020), a higher albumin level (3.8 vs 3.3 g/dL, *P* = .001), and a lower total bilirubin level (1.2 vs 1.6 mg/dL, *P* = .008) than the BCLC B/C group. The BCLC stage 0/A group showed a higher proportion of diabetes (34.6% vs 14.8%, *P* = .038) than the BCLC B/C group but a similar proportion of hypertension, cirrhosis, and fatty liver. Liver stiffness measurements by transient elastography and the controlled attenuation parameter were similar between the 2 groups.

The WBC count, platelet count, alpha-fetoprotein, AST, ALT, creatinine, PT INR, and total cholesterol level of the BCLC stage 0/A group were similar to those of the BCLC B/C group (10.9% vs 8.0%, *P* = .428).

The patients received initial treatment according to the BCLC criteria. Among the 223 patients, 196 patients received initial treatment for HCC at our center, while 27 patients wished to be transferred to another hospital. These patients received resection (n = 37), liver transplantation (n = 1), radiofrequency ablation (RFA, n = 25), transcatheter arterial chemoembolization (TACE, n = 126) or supportive care only (n = 7). No patients received systemic chemotherapy as their initial HCC treatment. Initial treatments for HCC are summarized in Table S1, Supplemental Digital Content, http://links.lww.com/MD/J453.

### 3.2. Baseline clinical characteristics of the patients with de novo HCC according to TNM staging

When classified according to the AJCC-TNM staging system, 154 patients (69%) were in stage I and 69 patients (31%) were stage II or higher. Ninety-two (41%), 65 (29%), 52 (23%), 5 (2%), 6 (3%), and 3 (1%) patients were diagnosed with HCC in AJCC-TNM stage IA, AJCC-TNM stage IB, AJCC-TNM stage II, AJCC-TNM stage IIIA, AJCC-TNM stage IIIB, and AJCC-TNM stage IV, respectively (Fig. 2).

The comparative results of the clinical features at diagnosis between patients with AJCC-TNM stage I HCC and those with stages II to IV HCC were investigated. The median age (70.5 vs 68.6 years, *P* = .201) and percentage of males (65.5% vs 95.6%, *P* = .058) in the AJCC-TNM stage I group and the AJCC-TNM stage II to IV group were not significantly different. There were similar proportions of patients with diabetes, hypertension, cirrhosis, and fatty liver disease between the 2 groups. Similarly, there were no significant differences in body mass index (BMI), liver stiffness measurements, or controlled attenuation parameter between the AJCC-TNM stage I and the AJCC-TNM stage II to IV group. The AJCC-TNM stage I group showed a higher albumin level (3.8 vs 3.5 g/dL, *P* = .007). In contrast, the WBC count, hemoglobin, platelet, AFP, AST, ALT, total bilirubin, creatinine, PT INR, and total cholesterol levels were similar between the 2 groups (Table 2).

**Table 2:** Baseline clinical characteristics of the study subjects (TNM stage).

Variables	Total (n = 223)	TNM stage I (n = 154, 69%)	TNM stage II–IV (n = 69, 31%)	*P* value*
Age (yr)†	70.0 (28.0–98.0)	70.5 (28.0–98.0)	68.6 (52.0–92.0)	.201
Sex (male), n (%)	152 (68.1)	101 (65.5)	66 (95.6)	.058
DM, n (%)	72 (32.2)	53 (34.4)	19 (27.5)	.469
Hypertension, n (%)	63 (28.2)	44 (28.5)	19 (27.5)	.908
LC, n (%)	167 (74.8)	115 (74.6)	52 (75.3)	.384
Fatty liver, n (%)	12 (5.3)	11 (7.1)	1 (1.4)	.097
CTP A/B/C n (%)	186/29/8 (83.4/13.0/3.6)	136/16/2(88.3/10.4/1.3)	50/13/6 (72.5/18.8/8.7)	.002
BMI (kg/m^2^)	24.7 (15.4–35.9)	24.9 (15.4–35.9)	24.0 (17.0–34.8)	.120
LS (kPa)	20.7(4.0–75.0)	21.4 (4.0–75.0)	18.5 (4.0–12.0)	.522
CAP (dB/m)	226.5 (125.0–337.0)	228.0 (125.0–337.0)	221.0 (188.0–274.0)	.773
WBC (1000/*µℓ*)	4696 (1600–10,030)	4670 (1600–9480)	4730 (2360–10,000)	.824
Hemoglobin (g/dL)	13.1 (6.4–17.7)	13.2 (6.4–17.7)	12.8 (8.5–16.7)	.157
Platelet (1000/*µℓ*)	111.6 (24.0–334.0)	108.3 (24.0–334.0)	119.2 (28.0–327.0)	.229
AFP (ng/mL)	22.0 (0.9–493.0)	25.7 (0.9–493.0)	13.0 (2.0–145.0)	.106
AST (IU/L)	52.6 (15.0–441.0)	49.7 (18.0–411.0)	59.6 (15.0–441.0)	.154
ALT (IU/L)	43.2 (5.0–511.0)	40.5 (5.0–511.0)	49.6 (10.0–298.0)	.181
Total bilirubin (mg/dL)	1.3 (0.2–5.3)	1.2 (0.2–4.5)	1.4 (0.3–5.3)	.157
Albumin (g/dL)	3.7 (2.0–5.2)	3.8 (2.0–5.2)	3.5 (2.3–4.9)	.007
Creatinine (mg/dL)	0.9 (0.5–3.5)	0.95 (0.5–3.5)	0.93 (0.7–1.5)	.732
PT, INR	1.2 (0.9–2.0)	1.2 (0.9–2.0)	1.3 (0.9–1.8)	.112
Total cholesterol (mg/dL)	152.1 (52.0–585.0)	154.7 (64.0–585.0)	145.8 (52.0–270.0)	.225

AFP = alpha-fetoprotein, ALT = alanine aminotransferase, AST = aspartate aminotransferase, BMI = body mass index, CAP = controlled attenuation parameter, CTP = Child–Turcotte–Pugh, DM = diabetes mellitus, INR = international ratio, LC = liver cirrhosis, LS = liver stiffness, PT = prothrombin time, WBC = white blood cell.

* *P* values were calculated using the *t* test or *chi*-square test.

† Median (range).

### 3.3. Univariate and multivariate logistic regression analyses for factors related to the advanced stage of HCC at initial diagnosis

Univariate and multivariate regression analyses were conducted to investigate the factors associated with an advanced stage of HCC at initial diagnosis, as listed in Table 3. In the univariate model, diabetes mellitus (odds ratio [OR] 0.311, 95% confidence interval [CI] 0.104–0.934, *P* = .037), CTP score (OR 2.841, 95% CI 1.504–5.368, *P* = .001), hemoglobin (OR 0.816, 95% CI 0.679–0.980, *P* = .030), total bilirubin (OR 1.699, 95% CI 1.114–2.591, *P* = .014), albumin (OR 0.406, 95% CI 0.225–0.733, *P* = .003), and PT INR (OR 9.448, 95% CI 1.724–1.778, *P* = .010) were significantly associated with a late-stage HCC at the initial diagnosis. However, the presence of cirrhosis (*P* = .712), BMI (*P* = .760), and sex (*P* = .482) had no significant effect on the diagnosis of late-stage HCC.

**Table 3:** Significant predictive factors of advanced HCC.

Variables	Univariate analysis HR (95%CI)	*P* value	Multivariate analysis HR (95%CI)	*P* value*
Age (yr)	1.023 (0.984–1.064)	.253		
Sex (male)	1.193 (0.498–2.857)	.692		
DM	0.311 (0.104–0.934)	.037	0.614 (0.301–0.925)	.073
Hypertension	0.660 (0.254–1.714)	.394		
LC	1.264 (0.485–3.296)	.631		
Fatty liver	0.000 (0.000)	.999		
CTP score	2.841 (1.504–5.368)	.001	1.547 (1.177–2.032)	.002
BMI	0.910 (0.794–1.042)	.173		
LS	0.749 (0.514–1.093)	.134		
CAP	0.993 (0.954–1.033)	.720		
WBC	1.018 (0.805–1.289)	.879		
Hemoglobin	0.816 (0.679–0.980)	.030	0.920 (0.743–1.138)	.440
Platelet	0.999 (0.992–1.006)	.796		
AFP	0.999 (0.989–1.009)	.793		
AST	1.005 (0.999–1.011)	.106		
ALT	1.003 (0.996–1.010)	.355		
Total bilirubin	1.699 (1.114–2.591)	.014		
Albumin	0.406 (0.225–0.733)	.003		
Creatinine	0.778 (0.162–3.735)	.753		
PT, INR	9.448 (1.724–51.778)	.010		
Total cholesterol	0.992 (0.981–1.002)	.114		

AFP = alpha-fetoprotein, ALT = alanine aminotransferase, AST = aspartate aminotransferase, BMI = body mass index, CAP = controlled attenuation parameter, CTP = Child–Turcotte–Pugh, DM = diabetes mellitus, HCC = hepatocellular carcinoma, INR = international ratio, LC = liver cirrhosis, LS = liver stiffness, PT = prothrombin time, WBC = white blood cell.

* For multivariate analysis, we selected DM, CTP score, and hemoglobin, but total bilirubin, albumin, and PT INR were not included.

In addition, multivariate analysis was performed to identify the risk factors for patients diagnosed with late-stage HCC. CTP score (OR 1.547, 95% CI 1.177–2.032, *P* = .002) was confirmed to be the only independent factor. Thus, patients with higher CTP scores, regardless of cirrhosis, were more likely to be diagnosed with HCC in the later stages.

## 4. Discussion

In this study, among the 223 patients initially diagnosed with HCC, 27 (13%) were diagnosed with BCLC stages B and C. According to the AJCC-TNM staging system, 69 patients (31%) had stages II, III, and IV disease. Patients with late-stage HCC showed higher CTP classifications B and C at initial diagnosis. CTP classification was the only independent factor for late-stage HCC at the initial diagnosis. Age, sex, presence of cirrhosis, and AFP level were not associated with delayed HCC diagnosis. Although HCC has a higher occurrence rate in males than females, sex was not a significant independent factor for late-stage HCC at initial diagnosis in this study.

Therefore, patients with late-stage HCC at the initial diagnosis showed poor liver function, either with or without cirrhosis. This outcome may have resulted from HCC occurring in non-cirrhotic livers. Although HCC typically develops in the liver with a cirrhotic background,^[13,14]^ approximately 20% of HCC cases arises in non-cirrhotic livers.^[15]^ In particular, NASH-related HCCs occur in both underlying cirrhotic and non-cirrhotic underlying livers. Compared to other liver disease etiologies, the NAFLD population has a much higher proportion of HCC in non-cirrhotic to cirrhotic livers.^[16]^ Grohmann et al^[18]^ suggested that obesity may cause NASH and HCC through different mechanisms, caused by a dissociation between the STAT-1 and STAT-3 activation pathways. In addition, NAFLD/NASH-associated HCC is frequently diagnosed at a late-stage, probably because of the decreased screening rates for HCC in this population.^[15]^ On the other hand, Chaudhary et al^[17]^ reported that although patients with surgically resected NAFLD/NASH-related HCC showed a larger tumor size than other etiologies of HCC, it did not result in poorer outcomes. Nevertheless, the prevalence of HCC caused by NASH/NAFLD or alcohol consumption is increasing and is expected to continue to increase.^[19]^ Hence, closer attention should be paid to this population.

The recent westernization of Korea’s dietary pattern has increased the incidence of HCC caused by NAFLD in the Korean population. The rising prevalence of metabolic liver disorders and better control of viral hepatitis raise the possibility that the etiology of HCC in South Korea is evolving.^[20]^ We hypothesized that BMI might be linked to a delayed diagnosis of HCC because individuals with NASH- or NAFLD-associated HCC tend to have a higher proportion of HCC arising from a non-cirrhotic liver background. Calle et al reported that the risk of mortality from HCC is 4.5 times higher in men with a BMI of 35 to 40 kg/m^2^ than in patients with normal body weight. Moreover, obesity, metabolic syndrome, and NAFLD account for up to 4-fold increase in HCC.^[21]^ Nevertheless, the findings of this study showed that BMI was not associated with the initial diagnosis of late-stage HCC.

Furthermore, although the Child-Pugh classification is widely used to evaluate liver function, CTP class B patients may include a wide range of conditions. The revised BCLC staging system recommends various treatment strategies for patients with CTP class B HCC. Similarly, ascites in CTP class A suggests poor prognosis, which should be considered when making individual treatment decisions.^[22]^

To the best of our knowledge, this is the first study to analyze the characteristics of patients with advanced HCC at the initial diagnosis in Korea. This study had some limitations. First, the socioeconomic status and educational levels of the patients may be important factors affecting their access to health care, which were not analyzed in this study because of a lack of availability. Second, AFP was the only tumor marker for HCC included in the study, and AFP-L3 or PIVKA-II levels were unavailable in the study population. Third, other etiologies of liver cirrhosis, such as primary biliary cirrhosis, autoimmune hepatitis, or Wilson’s disease, were not included in the study population. Fourth, only a small number of patients were investigated because only those initially diagnosed at our center were analyzed. Lastly, there were 675 patients who were followed up at our hospital for less than 1 year, and they were excluded from this study since they did not have sufficient data for analysis. non available data may be another limitation of our study. Further studies with more patients are warranted to investigate the characteristics of patients with late-stage HCC at initial diagnosis.

Blanc et al^[23]^ reported that between 2015 and 2017, more than 2 to 3rd of patients (68.8%) were in the late-stages of intermediate, advanced, or terminal stages at the time of HCC diagnosis. Only 16% of patients had previously undergone curative treatment, and more than half of the patients received only the best supportive care at the later stages of the intermediate, advanced, and terminal stages. Therefore, more rigorous surveillance is needed for these stratified patients at risk of developing late-stage HCC.

Unlike other cancers, the staging and prognosis of HCC depends on the TMN staging of the tumor and the patient’s liver function. This study showed that in patients with chronic liver disease undergoing regular screening for HCC, those with higher CTP scores were more likely to be diagnosed with HCC at a later stage. Patients who are diagnosed with HCC receive initial treatment such as resection, liver transplantation, RFA, TACE, systemic chemotherapy, or supportive care.^[5,24]^ The survival benefit of patients with BCLC stage B HCC will be increased by extending the indications for radical treatments and including these treatments as the first option.^[25]^

## 5. Conclusion

Patients with higher CTP scores are at a greater risk of HCC diagnosis at a later stage. Therefore, careful attention is needed for patients with high CTP scores during follow-up. These patients may require a shorter interval between visits and laboratory tests with advanced imaging modalities, such as CT, rather than ultrasonography.

## Author contributions

**Conceptualization:** Sangmi Jang, Young-Joo Jin, Jung Hwan Yu.

**Data curation:** Sangmi Jang, Jin-Woo Lee, Dam Kwon, Jung Hwan Yu.

**Formal analysis:** Sangmi Jang, Jin-Woo Lee, Dam Kwon, Jung Hwan Yu.

**Investigation:** Sangmi Jang, Young-Joo Jin, Jung Hwan Yu.

**Methodology:** Sangmi Jang, Young-Joo Jin, Jung Hwan Yu.

**Project administration:** Sangmi Jang, Young-Joo Jin, Jung Hwan Yu.

**Software:** Sangmi Jang, Jung Hwan Yu.

**Supervision:** Jin-Woo Lee, Jung Hwan Yu.

**Validation:** Dam Kwon, Jung Hwan Yu.

**Visualization:** Sangmi Jang, Jung Hwan Yu.

**Writing – original draft:** Sangmi Jang, Young-Joo Jin.

**Writing – review & editing:** Sangmi Jang, Jin-Woo Lee, Dam Kwon, Jung Hwan Yu.

## Supplementary Material


