# Supplementary material for: Risk factors associated with late hepatocellular carcinoma detection in patients undergoing regular surveillance
Source: Medicine (Baltimore). 2023 Aug 11;102(32):e34637. doi: 10.1097/MD.0000000000034637 (PMC10419803; doi:10.1097/MD.0000000000034637)
Supplement: Supplementary file 1 [file medi-102-e34637-s001.pdf]

Supplementary Table 1. Initial treatment for HCC according to BCLC staging criteria.

n (%)	Total (n=196)	BCLC 0 (n=69)	BCLC A (n=106)	BCLC B (n=16)	BCLC C (n=5)
Resection	37 (18.9)	9 (13.0)	27 (25.5)	1(6.3)	0 (0.0)
LT	1 (0.5)	0 (0.0)	1 (0.9)	0 (0.0)	0 (0.0)
RFA	25 (12.7)	8 (11.6)	17 (16.0)	0 (0.0)	0 (0.0)
TACE	126 (64.3)	51 (73.9)	57 (53.8)	15 (93.7)	3 (60.0)
Systemic chemotherapy	0 (0.0)	0 (0.0)	0 (0.0)	0 (0.0)	0 (0.0)
Supportive care only*	7 (3.6)	1 (1.5)	4 (3.8)	0 (0.0)	2 (40.0)

BCLC = Barcelona Clinic Liver Cancer, LT = liver transplantation, TACE = transcatheter arterial chemoembolization, RFA - radiofrequency ablation.

* 5 patients in BCLC 0 or A stages only received supportive care due to old age, poor general condition, or economic difficulty.
